# Divergent Manifestations in Biallelic Versus Monoallelic Variants of RP1-, BEST1-, and PROM1-Associated Retinal Disorders

**DOI:** 10.3390/ijms26146615

**Published:** 2025-07-10

**Authors:** Maximilian D. Kong, Jedrzej Golebka, Vanessa R. Anderson, Caroline Bao, Johnathan A. Bailey, Abdhel Exinor, Aykut Demirkol, Stephen H. Tsang

**Affiliations:** 1 Jonas Children’s Vision Care and Bernard & Shirlee Brown Glaucoma Laboratory, Institute of Human Nutrition, Columbia Stem Cell Initiative, New York, NY 10032, USA; mk5010@cumc.columbia.edu (M.D.K.); jg4713@cumc.columbia.edu (J.G.); vra2108@cumc.columbia.edu (V.R.A.); cb3997@cumc.columbia.edu (C.B.); jab2477@cumc.columbia.edu (J.A.B.); ae2647@columbia.edu (A.E.); ad3871@cumc.columbia.edu (A.D.); 2 Edward S. Harkness Eye Institute, Columbia University Irving Medical Center, New York-Presbyterian Hospital, New York, NY 10032, USA; 3 Downstate Medical Center, State University of New York, New York, NY 11203, USA; 4 Edward S. Harkness Clinical Coordinating Center, Columbia University, New York, NY 10032, USA; 5 Department of Ophthalmology, Vagelos College of Physicians and Surgeons, Columbia University Irving Medical Center, New York, NY 10032, USA; 6 Department of Pathology and Cell Biology, Vagelos College of Physicians and Surgeons, Columbia University Irving Medical Center, New York, NY 10032, USA

**Keywords:** inherited retinal disease (IRD), retinitis pigmentosa (RP), RP1 Axonemal Microtubule-Associated Protein (RP1), Bestrophin 1 (BEST1), Prominin 1 (PROM1), optical coherence tomography (OCT), autofluorescence, electroretinogram (ERG)

## Abstract

To compare the clinical characteristics of inherited retinal diseases (IRDs) caused by biallelic versus monoallelic variants in the *RP1*, *BEST1*, and *PROM1* genes. A total of 52 patients (26 female) with genetically confirmed IRDs were retrospectively selected from the records of the Harkness Eye Institute Clinical Coordinating Center at Columbia University Irving Medical Center. In *RP1*, 3 individuals with biallelic variants and 22 patients with monoallelic variants classified as pathogenic or likely pathogenic were selected. In *BEST1*, eight individuals with biallelic variants and nine individuals with monoallelic variants classified as either pathogenic or likely pathogenic were included. In *PROM1*, four individuals with biallelic variants and six patients with monoallelic variants classified as pathogenic or likely pathogenic were selected. All patients underwent multimodal retinal imaging and, when available, full-field electroretinography (ffERG). In all three genes, individuals with biallelic variants had markedly earlier disease onset and more severe phenotypes. In *RP1*, on SD-OCT, foveal involvement was observed in all biallelic cases (3/3, 100%) and in 4/22 (18%) monoallelic cases. In *BEST1*, the average age of onset in the biallelic cohort was 7.12 years, and the average age was 32.7 years in the monoallelic cohort. Four of eight (50%) patients in the biallelic group were additionally found to have widespread serous lesions outside of the central macula. This finding was not observed in the monoallelic group. Three of eight (38%) biallelic *BEST1* patients had moderate reductions in their photopic flicker. All monoallelic *BEST1* patients had photopic responses within the normal range. *PROM1* biallelic cases showed severe functional impairment on ffERG, while most monoallelic cases retained normal responses. In the biallelic cohort, four of four (100%) of patients had severely attenuated or extinguished photopic responses. In the monoallelic *PROM1* group, four of five (80%) monoallelic *PROM1* patients had normal photopic responses, and P2-2 had mildly attenuated photopic responses. Individuals with biallelic variants exhibited earlier disease onset, more severe retinal degeneration, and significantly reduced retinal function compared with those with monoallelic variants. These observations highlight the role of loss-of-function mechanisms in more aggressive disease courses and underscore the importance of considering zygosity when determining prognosis and planning gene-based therapies.

## 1. Introduction

Inherited retinal diseases (IRDs) are a group of disorders characterized by progressive dysfunction and degeneration of photoreceptors, often leading to severe vision impairment or blindness. IRDs include conditions such as retinitis pigmentosa (RP), cone–rod dystrophy (CORD), and macular dystrophy. IRDs affect approximately 1 in 3000 individuals worldwide and represent a leading cause of vision loss among working-age adults [1,2]. These disorders are genetically heterogeneous, with over 260 disease-associated genes identified to date [3]. The increased accessibility of genetic testing has enabled broader identification of causal mutations in IRD patients [4]. This has uncovered extensive phenotypic variability and disease severity, even among patients with mutations in the same gene [5,6,7,8]. This diversity of clinical manifestations is particularly evident in three key IRD genes: *RP1*, *BEST1*, and *PROM1*.

For *RP1*, mutations can cause either autosomal dominant (AD) or autosomal recessive (AR) forms of RP or CORD. It has been suggested that AD mutations cause disease in a dominant-negative manner, with mutations occurring within or immediately downstream of the BIF domain [9]. AR variants, on the other hand, result in loss-of-function (LOF) mutations from truncation of *RP1* before the BIF domain or in the N- or C-terminals [10].

*BEST1* mutations can cause Best vitelliform macular dystrophy, which follows an AD inheritance pattern, or AR bestrophinopathy. AD *BEST1* mutations can act via a dominant-negative, gain-of-function, or LOF mechanism, while AR mutations result in loss of protein function alone [11,12,13]. AR mutations occur throughout the *BEST1* gene, and AD and AR variants show minimal overlap [11].

Last, for *PROM1*, mutations can cause AD or AR CORD, AD macular dystrophy, or ARRP. AR diseases are associated with LOF mutations, and AD diseases are associated with a dominant-negative effect [14,15]. *PROM1* mutations are distributed across the entire gene [14].

Previous studies have observed that biallelic *RP1*, *BEST1*, and *PROM1* mutations were associated with more severe disease than monoallelic variants [14,16,17]. Specifically, biallelic mutations in *RP1* mutations are associated with earlier onset, worse visual acuity, and macular changes in the fundus [10]. In contrast, most patients with heterozygous *RP1* mutations develop ADRP with later onset of symptoms in the fourth decade of life [18]. Likewise, patients with recessive variants of *PROM1* began experiencing severe vision loss in the third decade of life, unlike those with AD mutations who maintained good vision into late adulthood [14]. Recessive *PROM1* mutations are also associated with widespread retinal dystrophy, whereas AD cases typically show dysfunction limited to the macula. In this study, we systematically compare clinical manifestations between monoallelic and biallelic variants in these three genes. We hypothesize that biallelic, loss-of-function variants will be associated with earlier onset and more severe retinal pathology than monoallelic variants.

## 2. Results

### 2.1. RP1

**Figure 1:**
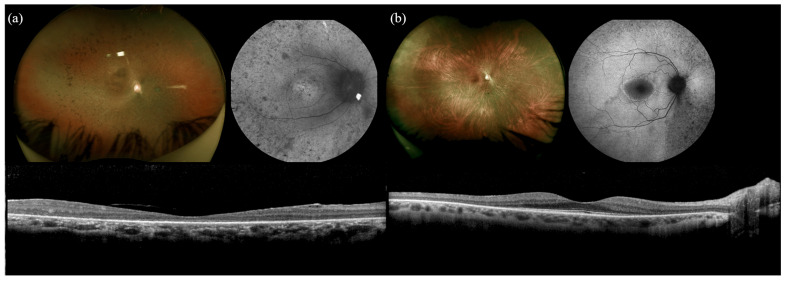
Multimodal imaging of a biallelic RP1 vs. monoallelic RP1 showing increased disease severity in the biallelic patient. R2-2, a 35-year-old South Indian female with biallelic mutations in RP1, was found to have severe thinning of the outer retinal layers, including involvement of the fovea (**a**). Patient R1-13, a 55-year-old White female with one mutation in RP1, has relative sparing of the ONL and EZ in the central macula (**b**).

The *RP1* cohort consisted of 3 individuals with biallelic pathogenic variants from 3 distinct families and 22 individuals with monoallelic pathogenic variants across 18 families. Clinical characteristics are summarized in Table 1. All symptomatic patients were diagnosed with retinitis pigmentosa (RP). Mean age of patient-reported symptom onset was 7.3 years in the biallelic group and 39.1 years in the monoallelic group. Nyctalopia was the most frequently reported symptom in both cohorts. Mean best-corrected visual acuity (BCVA) was 20/50 in the biallelic group and 20/30 in the monoallelic group. Fundus examination revealed classic RP features in all patients, including optic disc pallor, vascular attenuation, and intraretinal pigment migration. Three of three (100%) of the biallelic cohort were found to have CME on presentation, while 7/22 (32%) of the monoallelic cohort were found to have CME at presentation. On SD-OCT, all patients with biallelic mutations in *RP1* showed severe thinning of the outer retinal layers, including involvement of the fovea, suggesting a more severe progression of disease (Figure 1). In contrast, foveal involvement was detected in 4 of 22 (18%) monoallelic patients. There were two families in the monoallelic cohort that included patients who did not manifest disease while having a pathogenic mutation. In the family of R1-14, R-15, and R1-16, an affected mother had two unaffected daughters. In the family of R1-21 and R1-22, an affected father had one unaffected daughter. These unaffected children were found to have a normal examination, imaging, and full-field ERG (ffERG) testing.

ffERG was performed in all biallelic cases and 20 of 22 monoallelic cases. Among biallelic patients, two (R2-1 and R2-2) had extinguished photopic and scotopic responses, while R2-3 had extinguished scotopic and severely attenuated photopic responses. Patient R2-3 had extinguished scotopic responses and extremely attenuated photopic responses. These results indicate profound global photoreceptor dysfunction in biallelic cases. In the monoallelic group, 11 of 20 patients (55%) showed severely attenuated or extinguished responses, indicating a wider range of functional preservation.

### 2.2. BEST1

**Figure 2:**
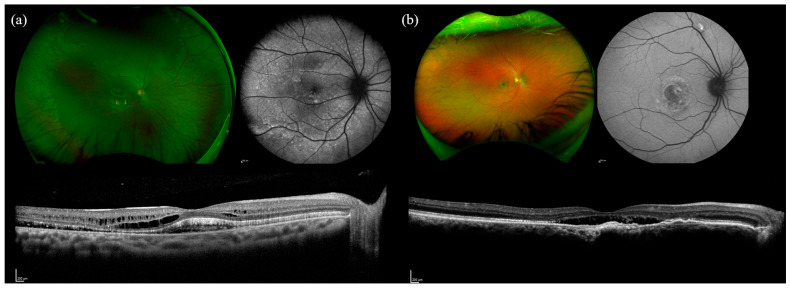
Multimodal imaging of an AR BEST1 vs. AD BEST1 showing increased disease severity in the patient with AR BEST1. B2-1, a 14-year-old Korean male with biallelic mutations in BEST1, was found to have widespread cystoid macular edema in addition to a central vitelliform lesion (**a**). Patient B1-1, a 57-year-old White male with one mutation in BEST1 (**b**). Despite additional decades of disease progression in B1-1, the peripheral retina remains unaffected.

The *BEST1* cohort comprised eight individuals from seven families with biallelic variants and nine individuals from eight families with monoallelic variants. Clinical characteristics are summarized in Table 2. The mean age of patient-reported symptom onset was 7.1 years in the biallelic group and 32.7 years in the monoallelic group. Decreased central visual acuity was the most frequently reported symptom in both groups. Mean BCVA was 20/40 in the biallelic cohort and 20/50 in the monoallelic cohort. Central vitelliform lesions were observed in both biallelic and monoallelic groups. All biallelic cases (eight of eight) exhibited vitelliform lesions extending beyond the macula. Additionally, four biallelic patients (50%) had widespread cystoid macular edema (CME) outside the central macula, a feature not observed in monoallelic patients (Figure 2).

ffERG was performed in six biallelic and four monoallelic patients. Three biallelic patients (38%) showed moderate reductions in photopic 30 Hz flicker responses. Scotopic responses were preserved in all biallelic cases. All monoallelic *BEST1* patients had photopic and scotopic responses within the normal range. Electro-oculography (EOG) testing was available in two biallelic patients and six monoallelic patients. Arden ratios for all patients were found to be between 1.5 and 1.

### 2.3. PROM1

**Figure 3:**
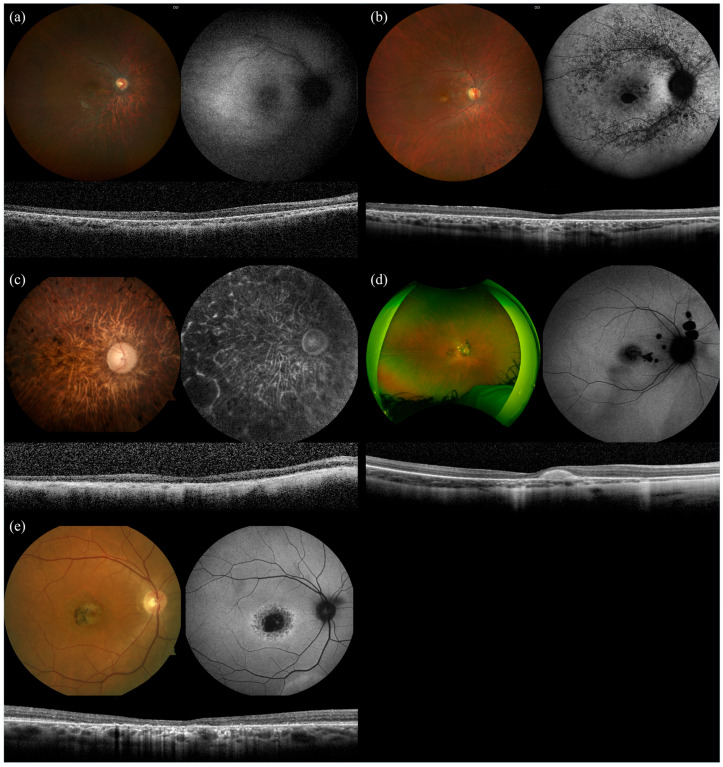
Multimodal imaging of biallelic PROM1 (P2-1, P2-2, P2-3, and P2-4) vs. monoallelic PROM1 (P1-5). Patients with biallelic variants showed younger age of onset and more severe characteristics on imaging and ERG. P2-1 (**a**), a 19-year-old male, was diagnosed with Leber congenital amaurosis. P2-2 (**b**), a 30-year-old female, was diagnosed with cone–rod dystrophy. P2-3 (**c**), a 61-year-old female, was diagnosed with retinitis pigmentosa. P2-4 (**d**), an 18-year-old female, was diagnosed with cone dystrophy. P2-5 (**e**), a 60-year-old male, was diagnosed with pattern macular dystrophy.

The *PROM1* cohort included four individuals from four families with biallelic variants and six individuals from six families with monoallelic variants. Clinical characteristics are summarized in Table 3. The mean age of patient-reported symptom onset was 9 years in the biallelic group and 37.3 years in the monoallelic group. Decreased central vision was the most frequently reported initial symptom in both groups. The mean BCVA was 20/200 in the biallelic group and 20/30 in the monoallelic group. Diagnoses in the biallelic group included retinitis pigmentosa (*n* = 3) and cone dystrophy (*n* = 1). In the monoallelic group, diagnoses included bull’s-eye maculopathy (*n* = 2), pattern macular dystrophy (*n* = 2), and nonspecific macular dystrophy (*n* = 3). Macular atrophy was commonly observed across both groups on fundus examination (Figure 3). Foveal atrophy on OCT was noted in all patients, regardless of zygosity.

All four biallelic *PROM1* patients and five monoallelic *PROM1* patients underwent ffERG. Among biallelic cases, two patients (P2-1 and P2-3) exhibited extinguished photopic and scotopic responses. P2-2 showed extinguished scotopic and severely attenuated photopic responses. P2-4, with cone dystrophy, demonstrated severely reduced photopic responses with preserved scotopic responses. In the monoallelic *PROM1* group, all patients retained normal scotopic responses. Four (80%) monoallelic *PROM1* patients had normal photopic responses, and P2-2 had mildly attenuated photopic responses.

## 3. Discussion

This study compared clinical phenotypes associated with biallelic and monoallelic variants in three IRD-associated genes traditionally linked to autosomal dominant inheritance: *RP1*, *BEST1*, and *PROM1*. In all cases, biallelic variants were associated with earlier onset, more extensive retinal degeneration, and greater functional impairment on ffERG. These findings suggest that the presence of a second pathogenic hit in these genes may result in a more severe phenotype.

AD *RP1* RP is known to have wide phenotypic variability, even among patients with the same mutation [19]. This was also seen in our cohort. In two families, children carrying the same pathogenic *RP1* variant as their affected parent remained clinically unaffected. These parents had late-onset disease with relatively mild clinical findings, suggesting age-related penetrance. Further follow-up is required to determine whether these adult-onset cases of *RP1* would have such a nondiagnostic exam earlier in life. These findings also support those of Nanda et al., who suggest that pathogenic *RP1* mutations exert a dominant negative effect rather than resulting in haploinsufficiency [20]. Biallelic *RP1* cases demonstrated a markedly more severe phenotype than monoallelic cases. All patients in the biallelic group had an earlier age of onset, dense pigment migration, and extremely poor ffERG responses. This suggested that the variability in phenotype in patients with biallelic mutations in *RP1* was relatively low compared with that in AD *RP1* RP. Patient R2-3 was found to be homozygous for a novel variant of uncertain significance, which was found to have a low allele frequency of 0.00003222 according to the gnomAD database supporting its pathogenicity (see Supplementary Table S1) [21]. In addition, in silico tools similarly predicted pathogenic changes to the protein sequence (CADD: 23.9) [22]. Furthermore, investigation of AR *RP1* RP patients found pathogenic mutations clustered in exon 4 between p.486 and p.635, which included the p.504 variant found in R2-3 [20]. This evidence in conjunction with R2-3’s clinical features provided evidence for pathogenicity. Overall, our findings of AR *RP1* patients exhibiting a more severe and homogenous phenotype involving an earlier age of onset, more severe imaging characteristics, and lower average ERG amplitudes are supported by other studies of biallelic *RP1* and provide important prognostic data for clinicians counselling these patients [20,23].

Compared with monoallelic *BEST1* cases, patients with biallelic variants had earlier onset and more widespread retinal involvement. Further, 30 Hz ERG amplitudes were below the normal range in half of the biallelic cohort, while the amplitudes in the monoallelic cohort were all within normal limits. These functional deficits may reflect the presence of widespread serous detachments and CME observed in the biallelic group. In addition, CME appeared refractory to standard carbonic anhydrase inhibitor therapy. These clinical findings are supported by previous studies that have found that AR disease is not restricted to the center and causes a diffuse RPE dystrophy [24,25]. In contrast with other studies of AR *BEST1*, we did not have biallelic patients report an adult onset of vision loss [24]. These changes may be explained by differing disease mechanisms. AD *BEST1* is thought to be caused by a dominant-negative effect, while AR *BEST1* is more likely to be LOF [11]. By history, all of the biallelic *BEST1* patients did not have affected family members, supporting the fact that these mutations are not pathogenic in the heterozygous state.

*PROM1* is noted to have a wide range of phenotypic manifestations even among biallelic and monoallelic patients [14]. Our findings of an early-onset panretinal degeneration are supported by previous studies of AR *PROM1* [14,15,26]. Our cohort included one patient, P2-2, who was found to be compound heterozygous for a pathogenic and VOUS in *PROM1*. Her VOUS, c.400C>G (p.Arg134Gly), has an extremely low allele frequency of 0.000001859, suggesting this variant is extremely rare in the general population (Supplementary Table S1) [21]. In silico predictors also predicted a high probability of pathogenicity (CADD: 27.9) [22]. Furthermore, variants at this position have been implicated in disease states including the p.Arg134Cys variant reported by Lee et al. [26]. The substitution of arginine with glycine represents a substantial physicochemical change, replacing a positively charged side chain with a small, nonpolar residue, which may disrupt local protein folding or extracellular interactions.

On ffERG, all biallelic *PROM1* patients had significant reductions in wave amplitude. Those with an RP phenotype were found to have nearly extinguished scotopic and photopic responses, while P2-3 had a diagnosis of cone dystrophy and had nearly extinguished photopic responses at a young age. In contrast, most monoallelic *PROM1* patients had somewhat reduced photopic amplitudes that did not fall outside of the normal range. This may represent an important distinguishing feature between these two phenotypes [27].

Limitations of this study include the retrospective nature of the study, limited sample size, and cross-sectional study design. Longitudinal data would provide more insight into the natural history and progression of disease in patients with biallelic versus monoallelic variants. The lack of available familial genetic testing also limited the ability to confirm segregation of variants.

Overall, biallelic variants in these genes conferred earlier, more severe structural and functional retinal degeneration than monoallelic variants. This observation is consistent with the established paradigm that autosomal recessive disease, particularly involving LOF mutations, typically results in more aggressive phenotypes than dominant-negative or haploinsufficiency-based disease.

Inherited retinal diseases (IRDs) caused by *RP1*, *BEST1*, and *PROM1* are traditionally classified as autosomal dominant; however, biallelic variants in these genes are increasingly identified through expanded genetic testing. These findings have important future implications. Understanding the mechanisms underlying these IRDs is important for developing gene therapies to reverse them. For autosomal dominant IRDs, potential for gene suppression has been demonstrated in mouse models of the *RHO* gene [28]. Recessive disorders have also been investigated using strategies such as gene supplementation and gene augmentation [29,30]. In addition to differences in treatment approaches, future clinical trials must also take phenotypic differences into account. Based on our results, patients who exhibit LOF mutations may have a smaller window for photoreceptor rescue. Patients with autosomal dominant disease rarely manifest with extinguished ERG responses. Our findings reinforce the clinical importance of genotypic context when evaluating IRD severity and progression. The distinction between monoallelic and biallelic disease is not only mechanistically relevant but may also influence the prognosis, timing of intervention, and eligibility for emerging therapies. As personalized approaches to retinal gene therapy continue to advance, these genotype–phenotype correlations will be essential for refining clinical trial design, optimizing patient selection, and ultimately improving outcomes.

## 4. Materials and Methods

### 4.1. Subjects and Inclusion Criteria

This was a retrospective, cross-sectional study of fifty-two individuals (26 female) identified from clinical records at Harkness Eye Institute Clinical Coordinating Center at Columbia University Irving Medical Center. To investigate *RP1*, 3 individuals with biallelic variants and 22 patients with monoallelic variants classified as pathogenic or likely pathogenic were selected. One individual in the biallelic *RP1* group carried a homozygous variant of uncertain significance (VOUS). For *BEST1*, 8 individuals with biallelic variants and 9 patients with monoallelic variants classified as pathogenic or likely pathogenic were included. To investigate *PROM1*, 4 individuals with biallelic variants and 6 patients with monoallelic pathogenic or likely pathogenic variants were selected; one biallelic patient harbored a compound heterozygous combination of one pathogenic variant and one VOUS. All variants were classified according to the American College of Medical Genetics and Genomics (ACMG) guidelines, and pathogenicity was supported using in silico prediction tools, including combined annotation dependent depletion (CADD) scores for protein-altering variants and SpliceAI scores for splice region variants [21,31]. Allele frequencies were obtained from the gnomAD v4.1.0 database to aid interpretation [21]. The age of onset was defined based on patient-reported age at first symptom. Informed consent was waived due to minimal patient risk and the retrospective study design, as approved by the Institutional Review Board (protocol AAAR8743). All procedures were reviewed and in accordance with the tenets of the Declaration of Helsinki.

### 4.2. Examination, Imaging, and Functional Testing

Ophthalmic examination involved measurement of best corrected visual acuity and pupil dilation with phenylephrine (2.5%) and tropicamide (1%). Dilation was followed by fundus examination, color fundus photography, spectral domain optical coherence tomography (SD-OCT), and fundus autofluorescence (FAF). SD-OCT was acquired using a Spectralis HRA + OCT (Heidelberg Engineering, Heidelberg, Germany). FAF was acquired using a Spectralis HRA + OCT, Optos 200Tx (Optos PLC, Dunfermline, UK), or Zeiss Clarus 700 (Carl Zeiss Meditec, Dublin, CA, USA). Digital color fundus and ultrawide-field color fundus photographs (Zeiss Clarus 700, Carl Zeiss Meditec, Jena, Germany; Optos 200 Tx, Optos PLC, Dunfermline, UK) were also obtained. ffERG was performed in house using Dawson, Trick, and Litzkow (DTL) electrodes and Ganzfeld stimulation on a Diagnosys Espion Electrophysiology System (Diagnosys LLC, Littleton, MA, USA) according to international standards [32]. ffERG photopic responses were categorized as within normal limits, mild attenuation, moderate attenuation, severe attenuation, or extinguished based on a clinician’s interpretation of a detectable waveform on the 30 Hz ERG with reproducible phase, and scotopic responses were based on a detectable wave form on the 0.01 dark-adapted ERG (Supplementary Table S2) [33,34] Electro-oculography (EOG) was performed in house using skin electrodes placed near the inner and outer canthi and Ganzfeld stimulation on a Diagnosys Espion Electrophysiology System (Diagnosys LLC, Littleton, MA, USA). Arden ratios were calculated using the light peak to dark trough in accordance with international standards [35].

## Figures and Tables

**Table 1:** Baseline clinical characteristics of patients with monoallelic and biallelic variants in RP1. Patients with biallelic variants showed younger age of onset and more severe characteristics on imaging and ERG. Clinical data include demographic information, genotype, age of onset and presentation, best-corrected visual acuity (BCVA), presenting symptoms, multimodal imaging findings, and full-field electroretinography (ffERG) responses. Unless otherwise specified, all clinical descriptions apply to both eyes. Genotype classifications include pathogenic (P), likely pathogenic (LP), and variants of uncertain significance (VOUS).

ID	Gender	Ethnicity	Variants	Age of Onset	Age at Presentation	BCVA-OD, OS	Initial Symptoms	Exam Findings	Peripheral HypoAF	Foveal Involvement on OCT	CME	ERG
R2-1	M	Cantonese	c.410C>A:p.Ser137Ter, P c.5017del:p.Tyr1673MetfsTer37, P	6	16	20/30, 20/40	Nyctalopia, DCV, photophobia, DPV	BEM, RPE mottling, rare pigment	Yes	Yes	Yes	P: Ext S: Ext
R2-2	F	South Indian	c.3843delT:p.Pro1282fs, P c.3843delT:p.Pro1282fs, P	7	31	20/100, 20/150	Nyctalopia	Macular atrophy, RPE mottling, rare pigment	Yes	Yes	Yes	P: Ext S: Ext
R2-3	F	Hispanic	c.1510T>G:p.Ser504Ala VOUS c.1510T>G:p.Ser504Ala VOUS	9	59	20/30, 20/50	Nyctalopia	Disc cupping, dense pigment	Yes	Yes	No	P: Severe Attenuation S: Ext
R1-1	M	Italian	c.1234dupA:p.Met412AsnfsTer7, P	25	55	20/30, 20/50	Nyctalopia	Peripapillary atrophy, moderate pigment peripherally, lacunae OD	Yes	No	Yes	P: Severe Attenuation S: Ext
R1-2	F	White	c.1498_1499del:p.Met500ValfsTer7, P	80	81	20/40, 20/40	DCV	Mild RPE mottling	No	No	No	P: Mild Attenuation S: Mild Attenuation
R1-3	M	White	c.2029C>T:p.Arg677Ter, P	28	7	20/20, 20/20	Nyctalopia, DCV	Rare pigment	Yes	No	No	P: Severe Attenuation S: Ext
R1-4	F	Lebanese	c.2029C>T:p.Arg677Ter, P	Unknown	84	20/40, 20/40	Nyctalopia, DPV	BEM, moderate pigment extending into the macula	Yes	Yes	Yes	P: Ext S: Ext
R1-5	M	Lebanese	c.2029C>T:p.Arg677Ter, P	28	54	20/25, 20/25	Nyctalopia, DPV	Moderate pigment nasally	Yes	Yes	Yes	P: Severe Attenuation S: Ext
R1-6	F	White	c.2029C>T:p.Arg677Ter, P	50	50	20/20, 20/25	Blind spots	Rare pigment nasally, no vessel attenuation	Yes	No	No	P: Mild Attenuation S: Moderate Attenuation
R1-7	F	White	c.2029C>T p.Arg677Ter, P	50	55	20/25, 20/20	DPV, nyctalopia	Rare pigment	Yes	No	No	P: Moderate Attenuation S: Ext
R1-8	M	Hungarian, French	c.2105_2108del:p.Ile702ThrfsTer10, P	34	51	20/20, 20/25	Decreased depth perception, DPV	BEM, moderate pigment	Yes	No	No	P: Severe Attenuation S: ND
R1-9	F	White	c.2105_2108del:p.Ile702ThrfsTer10, P	36	56	20/20, LP	Nyctalopia, DCV, DPV	Moderate pigment, retininotomy scar, proliferative vitroretinopathy OS	Yes	No	Yes	P: Severe Attenuation S: ND
R1-10	F	White	c.2172dup:p.Ile725AspfsTer4, P	33	35	20/20, 20/25	Nyctalopia, DCV, photosensitivity	Vessels normal, few pigment nasally	Yes	Yes	Yes	P: Mild Attenuation S: Moderate Attenuation
R1-11	M	Unknown	c.2219C>G:p.Ser740Ter, P	53	57	20/20, 20/25	DPV, nyctalopia	Dense pigment	Yes	No	Yes	P: Severe Attenuation S: ND
R1-12	M	White	c.2285_2289del:p.Leu762TyrfsTer17, P	Unknown	65	20/20, 20/20	Nyctalopia, DPV	Moderate pigment more prominent nasally	Yes	No	No	P: ND S: ND
R1-13	M	Unknown	c.2285_2289del:p.Leu762TyrfsTer17, P	20	70	20/25, 20/30	Nyctalopia, DPV, photophobia	Moderate pigment	Yes	No	No	P: Severe Attenuation S: Ext
R1-14	F	Irish, English	c.2285_2289del:p.Leu762TyrfsTer17, P	55	56	20/30, 20/30	Nyctalopia, floaters, occasional flashes	Rare pigment	No	No	No	P: Mild Attenuation S: Mild Attenuation
R1-15	F	Irish, English	c.2285_2289del:p.Leu762TyrfsTer17, P	None	29	20/20, 20/20	Asymptomatic	Normal appearing fundus	No	No	No	P: WNL S: WNL
R1-16	F	Irish, English	c.2285_2289del:p.Leu762TyrfsTer17, P	None	12	20/20, 20/20	Asymptomatic	Normal appearing fundus	No	No	No	P: WNL S: WNL
R1-17	M	White, not Hispanic	c.2285_2289del:p.Leu762TyrfsTer17, P	58	57	20/40, 20/30	DPV, increased nyctalopia	Choroidal nevus OD, moderate pigment	Yes	Yes	Yes	P: Moderate Attenuation S: Ext
R1-18	F	White	c.2285_2289del:p.Leu762TyrfsTer17, P	12	48	20/20, 20/20	Nyctalopia	Dense pigment	Yes	No	Yes	P: Severe Attenuation S: Ext
R1-19	F	Ireland, PR, Spain, Corsica	c.2285_2289del:p.Leu762TyrfsTer17, P	51	51	20/25, 20/30	DPV, nyctalopia	Temporal cobblestoning, rare pigment nasally	Yes	No	No	P: Mild Attenuation S: Mild Attenuation
R1-20	F	Unknown	c.2479G>C:p.Glu827Gln, P	19	44	20/30, 20/30	DPV, increased nyctalopia	Rare pigment	Yes	No	No	P: Severe Attenuation S: Ext
R1-21	M	Chinese	c.5017del:p.Tyr1673MetfsTer37, LP	33	43	20/20, 20/30	DCV	Rare pigment	No	No	No	P: Moderate Attenuation S: Ext
R1-22	F	Chinese	c.5017del:p.Tyr1673MetfsTer37, LP	None	10	20/20, 20/20	None	Normal appearing fundus	No	No	No	P: WNL S: WNL

Abbreviations: DCV, decreased central vision; DPV, decreased peripheral vision; BEM, bull’s-eye maculopathy; HypoAF, hypoautofluorescence; CME, cystoid macular edema; P, photopic 30Hz light-adapted ERG; S, scotopic 0.01 dark-adapted ERG; Ext, extinguished; WNL, within normal limits; ND, not done.

**Table 2:** Baseline clinical characteristics of patients with monoallelic and biallelic variants in BEST1. Patients with biallelic variants showed younger age of onset and subretinal retinal detachments (SRDs) extending past the macula. This table summarizes genetic, demographic, clinical, imaging, and electrophysiological data at presentation. BCVA is listed as Snellen equivalent for each eye. OCT findings detail the extent of subretinal detachment (SRD), cystoid macular edema (CME), and retinal thinning. ERG responses are categorized for both photopic (P) and scotopic (S) functions. EOG results are reported as Arden ratios in the right and left eyes. All clinical observations apply to both eyes unless otherwise noted.

ID	Gender	Ethnicity	Mutation	Age of Onset	Age at Presentation	Initial Symptoms	BCVA-OD, OS	Exam Findings	OCT Findings	ERG	EOG-OD, OS
B2-1	M	Korea	c.763C>T:p.Arg255Trp, P c.113T>G:p.Ile38Ser, LP	9	12	DCV	20/25, 20/25	Vitelliform lesions in the macula	SRD extending past the macula, widespread CME	P: mild attenuation S: WNL	1.4, 1.4
B2-2	M	Europe	c.302C>T:p.Pro101Leu, P c.313C>T:p.Arg105Cys, VOUS	5	9	DCV	20/25, 20/25	Central serous detachments	SRD extending past the macula	P: WNL S: WNL	Not done
B2-3	F	Irish, German, Greek, Scandinavian	c.140G>A:p.Arg47His, P c.454C>G:p.Pro152Ala P	7	8	DCV	20/20, 20/50	Serous vitelliform lesions, small cup	SRD extending past the macula	P: WNL S: WNL	Not done
B2-4	M	West Europe	c.475C>T:p.Gln159Ter, LP c.602T>C:p.Ile201Thr, LP	7	9	DCV	20/25, 20/30	Central serous detachments	SRD extending past the macula	P: ND S: ND	Not done
B2-5	F	West Europe	c.475C>T:p.Gln159Ter, LP c.602T>C:p.Ile201Thr, LP	6	6	DCV	20/25, 20/25	Central serous detachments	SRD extending past the macula	P: ND S: ND	Not done
B2-6	M	Irish, German, USA	c.602T>C:p.Ile201Thr, P c.602T>C:p.Ile201Thr, P	8	56	DCV	20/60, 20/60	Atrophic macular scar, central RPE island OD, macular atrophy OS	Subretinal fibrosis of the maculae, widespread retinal thinning	P: moderate attenuation S: WNL	1.4, 1.3
B2-7	M	West African	c.842TCT[2]:p.Phe283del, P c.842TCT[2]:p.Phe283del, P	6	8	DCV	20/80, 20/150	Large drusenoid deposits in the periphery	SRD extending past the macula, widespread CME	P: moderate attenuation S:WNL	Not done
B2-8	M	Puerto Rican	c.821C>G:p.Pro274Arg, P c.821C>G:p.Pro274Arg, P	9	14	DCV	20/50, 20/60	Cystic macular degeneration, subretinal exudates in arcades and nasally	SRD extending past the macula, widespread CME	P: WNL S: WNL	Not done
B1-1	M	Italian, Northern European	c.89A>G:p.Lys30Ar, P	7	54	DCV	20/70, 20/70	Yellow vitelliform lesions, intraretinal pigment	Central SRD	P: ND S: ND	1.1, 1.3
B1-2	M	White	c.727G>A:p.Ala243Thr, P	41	59	DCV	20/40, 20/30	Few intraretinal pigment migration, mottling	Central SRD	P: WNL S: WNL	Not done
B1-3	M	Irish, German, Welsh	c.253T>C:p.Tyr85His, P	37	53	DCV	20/80, 20/70	Vitelliform lesions in the macula	Central SRD	P: ND S: ND	Not done
B1-4	F	Irish	c.203A>G:p.Tyr68Cys, P	56	63	DCV	20/40, 20/40	Large vitelliform lesions	Central SRD	P: WNL S: WNL	1.3, 1.5
B1-5	M	Irish, German, Welsh	c.253T>C:p.Tyr85His, P	15	53	DCV	20/60, 20/30	SRD with subretinal fluid	Central atrophy with subretinal fibrosis OD, central SRD OS	P: ND S: ND	Not done
B1-6	M	Spain	c.727G>A:p.Ala243Thr P	35	54	DCV	20/100, 20/80	Central atrophy with serous detachment, temporal deposits, peripheral areas of atrophy	Central SRD	P: WNL S: WNL	1.2, 1.4
B1-7	M	Italian	c.727G>A:p.Ala243Thr, P	63	67	DCV	20/50, 20/40	Yellow vitelliform lesions centrally	Central SRD, subretinal fibrosis	P: ND S: ND	1.3, 1.2
B1-8	F	White	c.663T>G:p.Cys221Trp, P	5	30	DCV	20/30, 20/60	Central vitelliform macular lesions, temporal atrophy OS	Central SRD, subretinal fibrosis	P: ND S: ND	1.2, 1.4
B1-9	F	Italian	c.727G>A:p.Ala243Thr, P	35	66	DCV	20/60, 20/60	Central atrophy and serous detachment	Central SRD	P: WNL S: WNL	1.1, 1.2

Abbreviations: P, pathogenic; LP, likely pathogenic; VOUS, variant of uncertain significance; DCV, decreased central vision; CME, cystoid macular edema; SRD, serous retinal detachment; P, 30Hz photopic ERG; S, 0.01 scotopic ERG; WNL, within normal limits; ND, not done.

**Table 3:** Baseline clinical characteristics of patients with monoallelic and biallelic PROM1-associated retinal disease. Patients with biallelic variants showed younger age of onset and more severe characteristics on imaging and ERG. This table summarizes demographic, genetic, and clinical features including age of onset and presentation, initial symptoms, clinical diagnosis, visual acuity, fundus and OCT findings, and full-field ERG responses. Genotypic classifications include pathogenic (P), likely pathogenic (LP), and variants of uncertain significance (VOUS). All clinical descriptions apply to both eyes unless otherwise specified.

ID	Gender	Ethnicity	Variants	Age of Onset	Age at Presentation	Initial Symptoms	Diagnosis	BCVA-OD, OS	Exam Findings	Peripheral HypoAF	Foveal Involvement on OCT	ERG
P2-1	M	Korean	c.1877_1878del:p.Ile626fs, P c.139del:p.His47fs, P	2	11	Nystagmus, DCV	Leber’s congenital amaurosis	20/80, 20/70	Fine yellow dots in the periphery	Yes	Yes	P: Ext S: Ext
P2-2	F	English, Scottish	c.1579-1G>C:Splice acceptor, P c.400C>G:p. Arg134Gly, VOUS	23	28	DCV	Cone–rod dystrophy	20/30, 20/30	Macular atrophy, pigment in the macula and periphery	Yes	Yes	P: severe attenuation S: Ext
P2-3	F	Mediterranean	c.2362_2372del:p.Ile788GlufsTer26, P c.1455-1G>A:Splice acceptor, LP	7	56	Nystagmus, DCV	Retinitis pigmentosa	LP, LP	Extensive atrophy, pigment throughout the retina	Yes	Yes	P: Ext S: Ext
P2-4	F	White	c.1142-1G>A:Intronic, P c.1142-1G>A:Intronic, P	4	12	DCV	Cone dystrophy	20/50, 20/40	BEM	No	Yes	P: severe attenuation S: WNL
P1-1	F	Guangdong Province, Cantonese	c.1117C>T:p.Arg373Cys, P	52	53	Photosensitivity, decreased central vision	Bull’s-eye maculopathy	20/30, 20/30	BEM, fine deposits, ERM OD	No	Yes	P: WNL S: WNL
P1-2	M	White	c.2290A>T:p.Lys764Ter, P	31	33	DCV	Macular dystrophy	20/70, 20/70	BEM	Yes	Yes	P: mild attenuation S: WNL
P1-3	M	White	c.1117C>T:p.Arg373Cys, P	42	51	Central blind spot, nyctalopia, photosensitivity	Pattern macular dystrophy	20/40, 20/50	BEM, pigment in the macula and periphery	No	Yes	P: WNL S: WNL
P1-4	F	White	c.1117C>T:p.Arg373Cys, P	None	25	Asymptomatic	Macular dystrophy	20/25, 20/25	Optic nerve drusen, flecks surrounding the fovea, inferior/nasal pigment	No	Yes	P: WNL S: WNL
P1-5	M	Italian	c.303 + 2T>C:Splice donor, LP	31	32	DCV	Macular dystrophy	20/25, 20/25	Granular retina	No	Yes	P: WNL S: WNL
P1-6	F	Swiss, Mexican	c.303 + 1G>A:Splice donor, P	43	48	DCV	Bull’s-eye maculopathy	20/20, 20/20	BEM, surrounding granular deposits	No	Yes	P: ND S: ND

Abbreviations: DCV, decreased central vision; BEM, bull’s-eye maculopathy; HypoAF, hypoautofluorescence; ERM, epiretinal membrane; P, 30 Hz photopic ERG; S, 0.01 scotopic ERG; Ext, extinguished responses; WNL, within normal limits; ND, not done.

## Data Availability

The datasets generated during and/or analyzed during the current study are available from the corresponding author on reasonable request.

## Supplementary Materials

The following supporting information can be downloaded at: https://www.mdpi.com/article/10.3390/ijms26146615/s1.
