# Supplementary material for: Divergent Manifestations in Biallelic Versus Monoallelic Variants of RP1-, BEST1-, and PROM1-Associated Retinal Disorders
Source: Int J Mol Sci. 2025 Jul 10;26(14):6615. doi: 10.3390/ijms26146615 (PMC12294461; doi:10.3390/ijms26146615)
Supplement: Supplementary file 1 [file ijms-26-06615-s001.zip › Supplemental Table S1.pdf]

Supplemental Table S1. Summary of patients (IDs beginning with R, B, and P refer to individuals with variants in *RP1*, *BEST1*, and *PROM1*, respectively, and their corresponding variants. Variant classifications are listed acording to AMCG classification and include: pathogenic (P), likely pathogenic (LP), and variants of uncertain significance (VOUS). Allele frequencies from gnomAD v4.1.0 [25]. For in silico scores, Combined Annotation Dependent Depletion (CADD) scores were reported for variants that affect the protein sequence, and SpliceAI scores were reported for variants located in intronic or splice site regions [26, 35]. CADD and SpliceAI scores were obtained from gnomAD when available; missing data from the database are noted as "unavailable." Abbreviations: P = pathogenic; LP = likely pathogenic; VOUS = variant of uncertain significance; CADD = Combined Annotation Dependent Depletion.

ID	Variant	Variant Classification	Allele Frequency	In Silico Score
R2-1	c.410C>A:p.Ser137Ter	P	0.00000062	CADD: 24.6
R2-1	c.5017del:p.Tyr1673MetfsTer37	P	0.000001239	CADD: 22.2
R2-2	c.3843delT:p.Pro1282fs	P	0.000001239	CADD: 16.2
R2-2	c.3843delT:p.Pro1282fs	P	0.000001239	CADD: 16.2
R2-3	c.1510T>G:p.Ser504Ala	VOUS	0.00003222	CADD: 23.9
R2-3	c.1510T>G:p.Ser504Ala	VOUS	0.00003222	CADD: 23.9
R1-1	c.1234dupA:p.Met412Asnfs*7	P	0.00001239	CADD: 20.4
R1-2	c.1498_1499del:p.Met500Valfs*7	P	0.00001115	CADD: 27.8
R1-3	c.2029C>T:p.Arg677*	P	0.000004338	CADD: 33.0
R1-4	c.2029C>T:p.Arg677*	P	0.000004338	CADD: 33.0
R1-5	c.2029C>T:p.Arg677*	P	0.000004338	CADD: 33.0
R1-6	c.2029C>T:p.Arg677*	P	0.000004338	CADD: 33.0
R1-7	c.2029C>T p.Arg677*	P	0.000004338	CADD: 33.0
R1-8	c.2105_2108del:p.Ile702Thrfs*10	P	unavailable	unavailable
R1-9	c.2105_2108del:p.Ile702Thrfs*10	P	unavailable	unavailable
R1-10	c.2172dup:p.Ile725Aspfs*4	P	0.0000006196	CADD: 24.3
R1-11	c.2219C>G:p.Ser740*	P	unavailable	unavailable
R1-12	c.2285_2289del:p.Leu762Tyrfs*17	P	0.000001861	CADD: 25.2
R1-13	c.2285_2289del:p.Leu762Tyrfs*17	P	0.000001861	CADD: 25.2
R1-14	c.2285_2289del:p.Leu762Tyrfs*17	P	0.000001861	CADD: 25.2
R1-15	c.2285_2289del:p.Leu762Tyrfs*17	P	0.000001861	CADD: 25.2
R1-16	c.2285_2289del:p.Leu762Tyrfs*17	P	0.000001861	CADD: 25.2
R1-17	c.2285_2289del:p.Leu762Tyrfs*17	P	0.000001861	CADD: 25.2
R1-18	c.2285_2289del:p.Leu762Tyrfs*17	P	0.000001861	CADD: 25.2
R1-19	c.2285_2289del:p.Leu762Tyrfs*17	P	0.000001861	CADD: 25.2
R1-20	c.2479G>C:p.Glu827Gln	P	0.000007438	CADD: 23.2
R1-21	c.5017del:p.Tyr1673MetfsTer37	LP	0.000001239	CADD: 22.2

R1-22	c.5017del:p.Tyr1673MetfsTer37	LP	0.000001239	CADD: 22.2
B2-1	c.763C>T:p.Arg255Trp	P	0.00002726	CADD: 27.7
B2-1	c.113T>G:p.Ile38Ser	LP	unavailable	unavailable
B2-2	c.302C>T:p.Pro101Leu	P	0.00001549	CADD: 31.0
B2-2	c.313C>T:p.Arg105Cys	VOUS	0.000007742	CADD: 33.0
B2-3	c.140G>A:p.Arg47His	P	0.00001054	CADD: 16.6
B2-3	c.454C>G:p.Pro152Ala	P	0.00002323	CADD: 26.5
B2-4	c.475C>T:p.Gln159Ter	LP	0.00001486	CADD: 37.0
B2-4	c.602T>C:p.Ile201Thr	LP	0.0001481	CADD: 27.8
B2-5	c.475C>T:p.Gln159Ter	LP	0.00001486	CADD: 37.0
B2-5	c.602T>C:p.Ile201Thr	LP	0.0001481	CADD: 27.8
B2-6	c.602T>C:p.Ile201Thr	P	0.0001481	CADD: 27.8
B2-6	c.602T>C:p.Ile201Thr	P	0.0001481	CADD: 27.8
B2-7	c.842TCT[2]:pPhe283del	P	0.00001982	CADD: 19.6
B2-7	c.842TCT[2]:pPhe283del	P	0.00001982	CADD: 19.6
B2-8	c.821C>G:p.Pro274Arg	P	0.00001115	CADD: 24.9
B2-8	c.821C>G:p.Pro274Arg	P	0.00001115	CADD: 24.9
B1-1	c.89A>G:p.Lys30Arg	P	0.0000006196	CADD: 22.4
B1-2	c.727G>A:p.Ala243Thr	P	0.000001239	CADD: 29.2
B1-3	c.253T>C:p.Tyr85His	P	unavailable	unavailable
B1-4	c.203A>G:p.Tyr68Cys	P	0.000001239	CADD: 32.0
B1-5	c.253T>C:p.Tyr85His	P	0.000001239	CADD: 32.0
B1-6	c.727G>A:p.Ala243Thr	P	0.000001239	CADD: 29.2
B1-7	c.727G>A:p.Ala243Thr	P	0.000001239	CADD: 29.2
B1-8	c.663T>G:p.Cys221Trp	P	unavailable	unavailable
B1-9	c.727G>A:p.Ala243Thr	P	0.000001239	CADD: 29.2
P2-1	c.1877_1878del:p.Ile626fs	P	0.0000006196	CADD: 26.8
P2-1	c.139del:p.His47fs	P	0.000004338	CADD: 9.65
P2-2	c.1579-1G>C:Splice acceptor	P	0.00003382	SpliceAI: 0.990
P2-2	c.400C>G:p.Arg134Gly	VOUS	0.000001859	CADD: 27.9
P2-3	c.2362_2372del:p.Ile788Glufs*26	P	0.000001259	CADD: 32.0
P2-3	c.1455-1G>A:Splice acceptor	LP	0.0000006456	CADD: 34.0
P2-4	c.1142-1G>A:Intronic	P	0.00004283	SpliceAI: 0.990
P2-4	c.1142-1G>A:Intronic	P	0.00004283	SpliceAI: 0.990
P1-1	c.1117C>T:p.Arg373Cys	P	0.000001243	CADD: 10.6
P1-2	c.2290A>T:p.Lys764*	P	unavailable	unavailable
P1-3	c.1117C>T:p.Arg373Cys	P	0.000001243	CADD: 10.6
P1-4	c.1117C>T:p.Arg373Cys	P	0.000001243	CADD: 10.6

P1-5	c.303+2T>C:Splice donor	LP	unavailable	unavailable
P1-6	c.303+1G>A:Splice donor	P	0.00000248	SpliceAI: 0.630

Abbreviations: P = pathogenic; LP = likely pathogenic; VOUS = variant of uncertain significance; CADD = Combined Annotation Dependent Depletion.
