# Supplementary material for: Divergent Manifestations in Biallelic Versus Monoallelic Variants of RP1-, BEST1-, and PROM1-Associated Retinal Disorders
Source: Int J Mol Sci. 2025 Jul 10;26(14):6615. doi: 10.3390/ijms26146615 (PMC12294461; doi:10.3390/ijms26146615)
Supplement: Supplementary file 1 [file ijms-26-06615-s001.zip › Supplemental Table S2.pdf]

Supplementary Table S2. Full-field electroretinography (ffERG) wave amplitudes for 3.0 light-adapted 30 Hz flicker (photopic) and 0.01 dark-adapted b-wave (scotopic) responses in the cohort. Amplitudes are reported in microvolts (µV) for the right (OD) and left (OS) eyes, where available. "Extinguished" denotes nonrecordable waveforms. "Not Done" indicates the test was not available. *For B2-6, scotopic waveforms were not available, but the clinical report indicated responses were within normal limits (WNL).

ID	3.0 Light Adapted 30Hz ERG Amplitudes (OD, OS)	0.01 ERG b-wave Amplitudes (OD, OS)
R2-1	Extinguished, Extinguished	Extinguished, Extinguished
R2-2	Extinguished, Extinguished	Extinguished, Extinguished
R2-3	4.95, 2.91	Extinguished, Extinguished
R1-1	0.835, 1.98	Extinguished, Extinguished
R1-2	25, 31.12	67.79, 60.88
R1-3	7.817, 7.876	Extinguished, Extinguished
R1-4	Extinguished, Extinguished	Extinguished, Extinguished
R1-5	1.642, 1.385	Extinguished, Extinguished
R1-6	13.37, 20.36	32.27, 45.47
R1-7	8.512, 9.221	Extinguished, Extinguished
R1-8	2.88, 3.177	Extinguished, Extinguished
R1-9	3.01, Extinguished	Extinguished, Extinguished
R1-10	24.66, 36.72	Extinguished, Extinguished
R1-11	6.289, 2.149	Extinguished, Extinguished
R1-12	Not Done	Not Done
R1-13	1.716, 1.672	Extinguished, Extinguished
R1-14	58.02, 65.5	99.85, 92.1
R1-15	63.23, 90.75	156.9, 191.7
R1-16	58.8, 88.57	507, 519
R1-17	9.085, 10.84	Extinguished, Extinguished
R1-18	1.963, 2.676	Extinguished, Extinguished
R1-19	43.39, 43.73	108.2, 98.44
R1-20	4.502, 2.79	Extinguished, Extinguished
R1-21	3.611, 4.368	Extinguished, Extinguished
R1-22	59.53, 51.16	292.8, 261.9
B2-1	44.91, 38.36	158.9, 124.5
B2-2	96.35, 67.38	150.8, 177
B2-3	131.8, 136.5	681.2, 400.5
B2-4	Not Done	Not Done
B2-5	Not Done	Not Done
B2-6	40.37, 34.67	Reported WNL*
B2-7	31.677, 27.747	70.68, 97.61
B2-8	48.07, 49.58	90.09, 120.8
B1-1	Not Done	Not Done
B1-2	63.41, 52.52	176.9, 162.3
B1-3	Not Done	Not Done

B1-4	95.92, 75.31	216, 327.8
B1-5	Not Done	Not Done
B1-6	97.39, 77.39	254.1, 288.7
B1-7	Not Done	Not Done
B1-8	Not Done	Not Done
B1-9	62.22, 72.02	116.3, 108.5
P2-1	Extinguished, Extinguished	Extinguished, Extinguished
P2-2	7.311, 1.972	Extinguished, Extinguished
P2-3	Extinguished, Extinguished	Extinguished, Extinguished
P2-4	3.303, 3.748	146.2, 149.3
P1-1	35.61, 85.66	171.1, 143.2
P1-2	32, 33	180, 172
P1-3	52.21, 40.19	154.2, 242
P1-4	82, 101	121, 186
P1-5	87.9, 87.9	377.2, 427
P1-6	Not Done	Not Done
